# Can Social Semantic Web Techniques Foster Collaborative Curriculum Mapping In Medicine?

**DOI:** 10.2196/jmir.2623

**Published:** 2013-08-15

**Authors:** Cord Spreckelsen, Sonja Finsterer, Jan Cremer, Hennig Schenkat

**Affiliations:** ^1^Division of Knowledge-Based SystemsDepartment of Medical InformaticsRWTH Aachen UniversityAachenGermany; ^2^Deanery of Student AffairsFaculty of MedicineRWTH Aachen UniversityAachenGermany; ^3^Department of Anesthesiology and Intensive Care MedicineRWTH Aachen UniversityAachenGermany

**Keywords:** curriculum mapping, medical education, Semantic Web, Social Web

## Abstract

**Background:**

Curriculum mapping, which is aimed at the systematic realignment of the planned, taught, and learned curriculum, is considered a challenging and ongoing effort in medical education. Second-generation curriculum managing systems foster knowledge management processes including curriculum mapping in order to give comprehensive support to learners, teachers, and administrators. The large quantity of custom-built software in this field indicates a shortcoming of available IT tools and standards.

**Objective:**

The project reported here aims at the systematic adoption of techniques and standards of the Social Semantic Web to implement collaborative curriculum mapping for a complete medical model curriculum.

**Methods:**

A semantic MediaWiki (SMW)-based Web application has been introduced as a platform for the elicitation and revision process of the Aachen Catalogue of Learning Objectives (ACLO). The semantic wiki uses a domain model of the curricular context and offers structured (form-based) data entry, multiple views, structured querying, semantic indexing, and commenting for learning objectives (“LOs”). Semantic indexing of learning objectives relies on both a controlled vocabulary of international medical classifications (ICD, MeSH) and a folksonomy maintained by the users. An additional module supporting the global checking of consistency complements the semantic wiki. Statements of the Object Constraint Language define the consistency criteria. We evaluated the application by a scenario-based formative usability study, where the participants solved tasks in the (fictional) context of 7 typical situations and answered a questionnaire containing Likert-scaled items and free-text questions.

**Results:**

At present, ACLO contains roughly 5350 operational (ie, specific and measurable) objectives acquired during the last 25 months. The wiki-based user interface uses 13 online forms for data entry and 4 online forms for flexible searches of LOs, and all the forms are accessible by standard Web browsers. The formative usability study yielded positive results (median rating of 2 (“good”) in all 7 general usability items) and produced valuable qualitative feedback, especially concerning navigation and comprehensibility. Although not asked to, the participants (n=5) detected critical aspects of the curriculum (similar learning objectives addressed repeatedly and missing objectives), thus proving the system’s ability to support curriculum revision.

**Conclusions:**

The SMW-based approach enabled an agile implementation of computer-supported knowledge management. The approach, based on standard Social Semantic Web formats and technology, represents a feasible and effectively applicable compromise between answering to the individual requirements of curriculum management at a particular medical school and using proprietary systems.

## Introduction

### Background

Curriculum mapping supports teachers, learners, and curriculum administrators by providing a comprehensive overview of a curriculum and its elements and their interrelations. It answers questions like “Where do we teach what?” [1]. Curriculum mapping is considered to be a demanding, data-intensive, and essentially collaborative effort [2]. The number of custom-built software platforms for curriculum mapping indicates a lack of available tools and standards [3]. Social Semantic Web (SSW) approaches combine Web content, which can be partly “understood” (ie, processed in a semantically sound way) by computer programs with social software, enabling the collaborative creation and maintenance of content to take place. Thus, SSW approaches provide promising solutions for implementing and maintaining curriculum mapping in medicine as a knowledge management process.

The revision of the national German medical licensing regulations enacted in 2002 enabled markedly changed medical curricula to be introduced at German medical schools, and this included the creation of a number of reform model curricula [4]. This change process increased the need for computer-supported curriculum mapping.

### Curriculum Mapping

In the 1970s, Hausman coined the term curriculum mapping in the context of curriculum planning [5]. Describing the differences between the prescribed, the taught, and the tested curricula (ie, the ideal curriculum as planned in advance, the curriculum delivered by the teachers, and the curriculum learned by the students), English then proposed curriculum mapping as an approach to realign these three “circles”, mainly by capturing the taught curriculum and comparing it to the ideal one [6]. Gjerde stressed the potentially positive effect of curriculum mapping on the congruence of learning objectives and tests used for evaluation [7].

In his comprehensive review of curriculum mapping, Harden recommended the approach as a pivotal factor in fostering coordination and communication of medical curricula [8]. He also emphasized the aspect of depicting the components of a curriculum and their interrelations explicitly in a curriculum map, which should provide different views (“windows”) focusing on, for example, the content, the learning events, or the learning resources of a given curriculum.

In 2008, a survey on the status of curriculum mapping in Canada and the United Kingdom found that 55% of the responding medical schools were in the process of establishing a curriculum map [3]. Notably, Willett also found that curriculum mapping was considered to be an ongoing process requiring “continual upgrading and maintenance”. Following their analysis, the large quantity of custom-built software indicates a shortcoming of available IT tools that meet the requirements of curriculum management.

### Computer-Supported Curriculum Management

The Faculty of Medicine at McGill University implemented an electronic curriculum map at an early stage; this allows a curriculum inventory to be performed [9]. Tufts Health Science Database (HSDB) was an early attempt to integrate content delivery and curriculum management. Lee et al describe the positive effects of HSDB on faculty development, curricular reform, and interdisciplinarity [10]. CurrMIT serves as a means to capture, manage, and compare the curricula of North American medical schools [11]. Curriculum mapping tools have also been developed in other health-related areas, for example, in the context of nursing education [12].

In contrast to the awareness of the role of electronic curriculum maps for innovative curriculum management and the early attempts to integrate learning content, many approaches—especially in German-speaking countries—focused on creating online learning objectives catalogs or databases. The Swiss Catalogue of Learning Objectives (SCLO) [13] was one of the most influential European projects for defining and managing structured, outcome-oriented learning objectives. SCLO provides an open-access Web portal that allows the objectives to be filtered by keyword, type, discipline, topic, and competence levels. In the meantime, LO catalogs similar to the SCLO have been implemented at various German medical schools [14-16]. The projects grant Web-based access to their users. All Web platforms are custom-built, which corresponds to the general trend found by Willett at UK and Canadian medical schools [3]. The online tool used for the Heidelberg Catalogue of Learning Objectives (HCLO) focuses on enabling interactive maintenance and improvement of the catalog. The Charité University Hospital, Berlin, uses its platform to support systematic curriculum mapping, and claims to be one of the first German faculties to do so. Hege et al similarly advocated the use of computer-based LO catalogs in the process of managing and mapping a curriculum [15,16]. In addition to catalogs of learning objectives, which address the complete curricula of medical schools, some approaches concentrate on a given medical specialty or field [17].

### Second-Generation Curriculum Management Systems

Watson et al distinguished a first from a second generation of curriculum management systems: the first generation comprises electronic curriculum maps and databases that support administrative processes, whereas the second generation is represented by “comprehensive knowledge-management systems primarily designed to support students and teachers in the learning and teaching process while also supporting administrative processes” [18].

eMed, developed at the University of New South Wales, is reported to be an integrated second-generation curriculum management system, combining various tools and services. eMed supports very different aspects of curriculum management, ranging from curriculum mapping to student portfolios, and bridging the gap between organizational, curricular, and economic needs [18]. Bell et al reported on a curriculum-mapping project that concentrates especially on curricular quality management by systematic curriculum review; this fosters integration, transparency, and communication [19]. The authors found the curriculum mapping process to be resource-intensive, and underlined the fact that none of the existing software tools designed for supporting curriculum mapping fully met the requirements of their project. Just recently, the University of Toronto developed CMap, a special computer-based curriculum-mapping tool that was able to detect an uneven distribution of teaching time with respect to the major topics and skills of a given planned curriculum [20].

### Semantic Indexing

An important prerequisite for enabling curriculum mapping and comprehensive curriculum management, based on databases organizing curricular data and learning objective catalogs, is semantic indexing. Learning objectives need to be retrievable by medical topic, similar learning content should be associated, and the coverage of learning objectives by learning events or examinations needs to become visible.

Denny et al investigated the ability of a text mining and classification tool to give an automatic estimate of the coverage of medical topics by lectures, based on texts documenting these lectures [21]. The approach proved successful in supporting semantic indexing of curricular events and outperformed, in that curricular context, a similar tool (MetaMap) that had primarily been designed for indexing scientific publications [21,22]. The taxonomy TIME (Topics for Indexing Medical Education) served as a means to index the elements of a curriculum map uniformly [23]. Thus, TIME enabled topic specific views, which show the contribution of curricular elements to specific outcomes. Dexter et al reported on using the US Medical Licensing Examination Step 1 Content Outline (USMLE Step1 CO) as a basis for indexing LOs in order to assess the completeness of topic coverage [24]. Instead of using preexisting taxonomies, semantic indexing can also be achieved by collaboratively created taxonomies, which evolve during the use of a given information system, so-called folksonomies. Gasevic et al reported an exemplary application of folksonomies to the maintenance of learning environments [25].

### Aachen Catalogue of Learning Objectives

In 2003, the Medical Faculty of the RWTH Aachen University started the Aachen Medical Model Curriculum (AMMC). The curriculum implements a spiral-shaped education process (“spiral curriculum” [26]) and aims at an early integration of preclinical and clinical education. In the second and third years, 11 multidisciplinary modules, each focusing on a particular organ system, form the backbone of the curriculum. The AMMC follows an explicit mission statement and defines learning goals for all modules. Furthermore, a faculty-spanning consensus process, which took place before the start of the model curriculum, defined the content of all modules. Nonetheless, the documentation of learning objectives merely relied on item lists, which proved insufficient for maintaining and further developing the curriculum. Thus, in 2011, the faculty formally decided to implement a comprehensive, Web-based catalog of competency-based learning objectives (Aachen Catalogue of Learning Objectives or ACLO). The spiral curriculum and multidisciplinary modules create challenging requirements concerning the elicitation, revision, and communication process of the LOs.

### Aims

The Web-based Aachen Catalogue of Learning Objectives (ACLO-Web) aims to support the implementation, maintenance, and use of the ACLO by establishing a Web-based knowledge management system based on Social Semantic Web technology and standards.

## Methods

### Elicitation and Revision Process of the ACLO

The faculty formed a special task force (“Learning Objectives Working Group”) in order to organize the implementation of the ACLO. The dean asked all contributing units (clinics, institutes, and external departments) to name representatives responsible for the detailed specification of LOs. Each representative needed to participate in a training program: mainly a 1-day workshop by trainers with certified competency in medical education. After the training, the representatives started the elicitation and specification of LOs based on predefined forms, while consulting everyone in their unit (clinic or institute) who was involved in teaching. In order to guarantee a high and equal level of data quality, especially with respect to thematic indexing of the LOs, a team of 1 medical expert and 2 student coworkers (the “ACLO Team”) was responsible for the primary data entry into the central catalog. This team continuously checked and improved the assignment of LOs to medical topics, curricular modules, and a responsible faculty. Furthermore, they supervised quality standards concerning the formulation of competency-based learning objectives. During the ACLO revision, the responsible representative, who could of course involve his or her colleagues, checked and eventually improved the LO specification.

When the Learning Objectives Working Group finally approved the LOs, the catalog was made accessible to students. Faculty members and students now continuously check the LO catalog for inconsistencies and monitor whether the LOs are adequately addressed by the courses and lectures. Their feedback should lead to the future improvement of the catalog.

### Requirements

An information system supporting the process described below has to meet the following requirements:

- Collaborative: The system needs to support a collaborative effort. It needs to provide decentralized access to a central LO repository, allow the storage and tracking of the whole history of changes made to the LOs in the collection (ie, support a versioning mechanism), and manage user accounts and roles.
- Structured: The system must allow the structured entry and retrieval of information; in order to enable a systematic search of different aspects of the LO specification, the system needs to store structured information (attribute-value pairs of the different aspects of the LO specification), which can be entered in online forms.
- Flexible: The system needs to be flexible with respect to changes in the representation, retrieval, and presentation of LOs. Ideally, the system should support the implementation process of the LO catalog from the very beginning. As a consequence of the ongoing implementation process, it should be possible to adapt the system to changing requirements.

### Social Semantic Web-Based Curriculum Management

The online catalog of learning objectives is based on Social Semantic Web technology, in order to support collaborative knowledge management flexibly. The project reported here uses MediaWiki (the software platform of Wikipedia) enhanced by additional software modules (extensions). We adopted the Semantic MediaWiki (SMW) and Semantic Forms extensions.

A MediaWiki application enables the collaborative maintenance of Web pages; the pages can easily be edited and linked by wiki users using only a Web browser. Wiki users can revisit or even undo all changes to a page logged by the system in the page history. Similar content can be presented uniformly by using wiki templates combining predefined text fragments with new information entered via variables. Comments can be added to existing wiki pages. MediaWiki provides different approaches for managing user accounts and granting access. At present, read access to ACLO requires a general password communicated to all students and teachers. Readers can also enter comments and annotations (reader role). ACLO grants write access (ie, the right to change the core content), to registered users only (author role). The creation of user accounts is restricted to the administrators of ACLO.

As a first step toward equipping the wiki pages with computer-readable semantics, pages can be assigned to categories, and pages addressing similar concepts can be organized together (eg, the categories “disease” and “symptom” may be defined for pages addressing diseases and symptoms, respectively). Based on such categories, MediaWiki automatically generates index pages.

**Figure 1:**
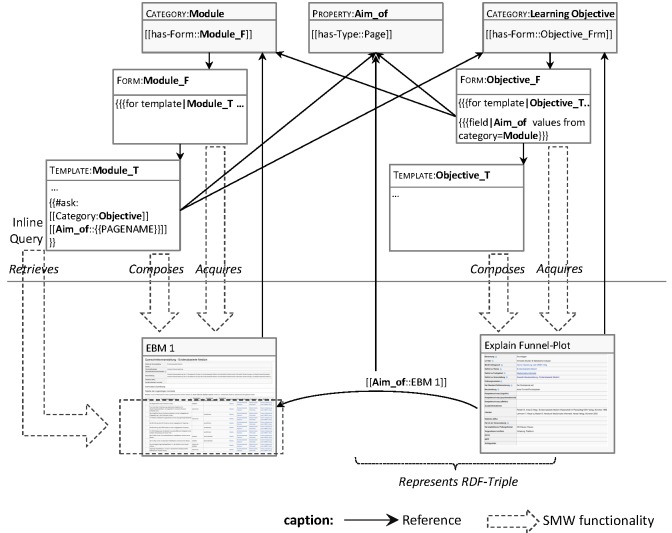
The SMW-based approach to the representation and acquisition of curricular information, illustrated by an example taken from ACLO-Web; the diagram includes abbreviated SMW syntax in order to illustrate why the references between the different building blocks are established.

The SMW extension [27] adds semantic relations (SMW properties) to the MediaWiki, allowing pages to be annotated by structured property-value pairs and associated by meaningful relations. A page describing a learning objective may refer (link) to a different page describing a teaching module by the property “Aim_of” (syntactically achieved by inserting the SMW annotation “[[Aim_of::*target-page*]]” on the referring wiki page) (see Figure 1). SMW properties form subject-predicate-object triples (referring page, property, referenced page, or value) as introduced by the Semantic Web approach [28]. The enhanced representational means of SMW directly correspond to a Web Ontology Language (OWL) based knowledge representation: each SMW page represents an (abstract) individual, SMW properties correspond to OWL properties, and wiki categories to OWL classes, respectively. In general, SMW annotations can be translated—and actually exported—to OWL DL statements (in OWL/RDF encoding), where OWL DL is an OWL sublanguage formally based on complete and decidable description logics [29,30]. Only some built-in SMW properties lack direct equivalents in OWL and are thus treated as annotation properties [27]. According to the above correspondence, most SMW annotations represent assertional knowledge, that is, they state facts concerning attributes of domain objects and their mutual relationships (A-Box statements of an ontology-based knowledge representation). Furthermore, but to a lesser extent (see below), SMW is able to represent statements defining domain concepts, for example, concerning classes of objects and admissible values of object attributes belonging to a given class, thus forming a conceptual schema of a domain (T-Box statements). Some SMW applications exploited this further by importing exiting ontologies.

SMW also defines an elaborated query syntax operating on its semantic annotations. SMW pages may include inline queries enabling the dynamic and consistent updating of facts throughout the wiki application. Furthermore, it is possible to not only query, but also to manage an SMW application based on a Resource Description Framework database (RDF-Triple Store) in combination with the SPARQL Protocol and RDF Query Language (SPARQL) [27,31].

SMW properties may also refer to primitive data types, such as strings, numbers, or dates, allowing pages to organize structured data instead of containing unstructured text. The Semantic Forms extension then supports structured data entry by defining forms. Thus, forms can foster structured and uniform data entry for all pages of a given category. When authors enter content via semantic forms, the created wiki pages are categorized automatically. Semantic forms offer drop-down lists, check boxes, and a sophisticated auto-completion mechanism, and allow data entry to be restricted to semantically sound values. As shown in Figure 1, the approach relies mainly on semantic forms when imposing schema constraints. While it is possible to define all categories and properties necessary for representing a given field, the declaration of a property can use only very general data types and does not allow its values to be restricted to a domain defined in terms of existing categories (as a T-Box of a domain ontology would do). The semantic forms are the means available for imposing the respective constraints. Figure 1 shows the SMW mechanisms imposing semantic constraints and enabling structured data management; each SMW category can use a semantic template for uniformly composing a page out of structured parameters and each template can make use of semantic forms allowing controlled data entry by online forms. Most notably, a template can include query syntax that dynamically and individually refers to the content of each page composed by that template.

### Core Data Model

**Figure 2:**
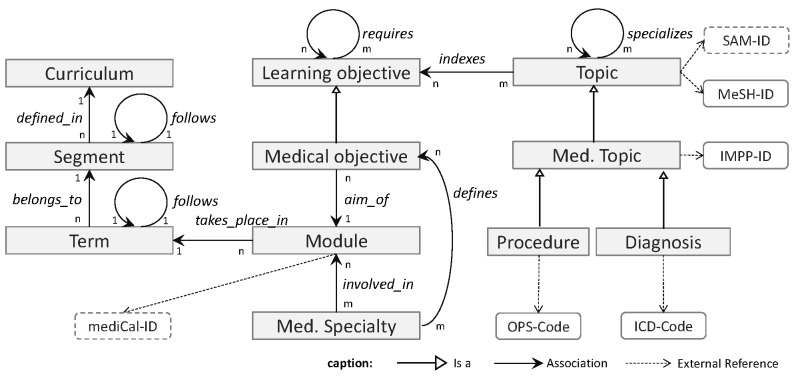
Core data model of ACLO-Web (grey boxes correspond to SMW categories, arrows correspond to SMW semantic relations; arrow subscripts (1-1, 1-n, n-m) correspond to one-to-one, one-to-many, and many-to-many relationship types, respectively; and boxes with rounded corners indicate references to external classification systems established by ID fields).

Figure 2 shows the core data model of the LO catalog. The diagram focuses on the most relevant aspects: each LO needs to be assigned to teaching modules of the curriculum (ie, courses, lectures, practicals, etc). Additionally, an LO needs to be characterized by the medical topic addressed by the LO and the medical specialty involved. The left-hand area of the diagram represents the curricular context (given by “Term”, “Segment”, “Curriculum”). The regulations of the course of study and the examinations define a planned curriculum. As official regulations are revised from time to time, teaching modules may be redefined or moved to different semesters. Thus, the catalog can reuse LOs in different modules following different regulations by assigning them to modules belonging to different versions of the curriculum.

Assigning a medical specialty to a teaching module implies that the respective clinic or institute is responsible for teaching (is “involved_in”) the multidisciplinary module. The direct relation “defines” indicates (institutional) authorship of medical objectives, which are learning objectives specifically addressing medical topics. The vast majority of LOs of ACLO belong to this type and are authored by teachers affiliated with a clinic or institute. The remaining LOs (some more general learning goals) were defined by conferences. These LOs are not associated with specific institutions.

The model represents medical topics (eg, diseases, therapeutic approaches, or professional skills) as independent entities. Therefore, LOs can be assigned to a set of medical topics, which nonetheless may be revised or extended during the implementation process of the catalog. At present, all authors of learning objectives can enter new topics, while an auto-completion function reduces the risk of entering syntactic variants and fosters the user’s awareness of existing topics. Users can also form hierarchic or associative links between topics. Thus, the set of available topics and its structure evolves due to collaborative effort and, therefore, forms a folksonomy. The assignment of topic to LOs is carried out manually. It complements the automatic categorization of wiki content by the semantic forms. The use of a controlled set of topics promotes consistent semantic indexing and retrieval of LOs. The model also indicates references to external information. By referencing a MeSH-ID, an ICD-Code, an OPS-Code, or an IMPP-ID, the topics are linked to established medical classifications. These references establish links to the Medical Subject Headings (MeSH), the International Classification of Diseases (ICD), the German modification of the International Classification of Procedure in Medicine (Operationen- und Prozedurenschlüssel - OPS), and the catalog of the Institute for Medical and Pharmaceutical Examination Items (IMPP), the national provider for final medical examinations, respectively. The system therefore allows it to be determined which requirements of the final examinations are covered by the learning objectives of a given module, term, and the whole curriculum, respectively. Additionally, the indexing of learning objectives by topics allows similar learning objectives of a given topic to be retrieved, which is achieved at present by using SMW subqueries within the system.

Additionally, the model provides 2 interfaces to different applications established at the medical school: (1) the SAM-ID leads to a semantic network used to index learning media produced by the local center for audiovisual media, and (2) the mediCal-ID links to the online calendar and room management system used for planning the terms. Both systems will be further integrated in the near future.

### Specification of Learning Objectives

The assignment of LOs to teaching modules, topics, and responsible specialties (treated as independent model entities) is complemented by a detailed specification of each LO. Roughly, this specification includes (1) the essential description of the LO, (2) further indexing, and (3) supplementary didactic information.

The essential LO description formulates the observable behavior of the students, showing the level of competence required by the LO. Each LO description begins with a text introduction, which is chosen from a given set of templates (eg, “The students are able to...”, “At least the top 10% of the participants can...”). The sentence is continued by free text describing the required behavior and then finished by a verb (to be selected out of a given set) indicating the level of competence (eg, “explain”, “show”, “appraise”), in a sequence matching German grammar. Semantic indexing is enabled by assigning predefined topics as described above. Supplementary didactic information allows an LO to be associated with other LOs that are named as prerequisites for achieving the LO in question (predecessor LOs). It is also possible to indicate recommended learning and teaching formats for the LO (eg, “Lecture”, “Problem-based learning”) and recommended assessment formats (eg, “Multiple choice test”, “Objective structured practical examination”).

### Global Consistency Checking

SMW technology enables structured data entry, semantic queries, and the dynamic consistent update of a large hypertext. Nonetheless, due to the relatively weak schema constraints imposed by the definition of categories and the semantic properties, complex or global semantic consistency criteria cannot be enforced algorithmically. As an example, the SMW platform alone provides no means for checking if an LO that is declared to be a prerequisite of a given LO is associated with prior learning events. In addition, complex cardinality constraints cannot be imposed on the SMW properties (eg, “There should be exactly one study section in each curriculum that has no predecessor”; in what follows, this is referred to as C1).

**Figure 3:**
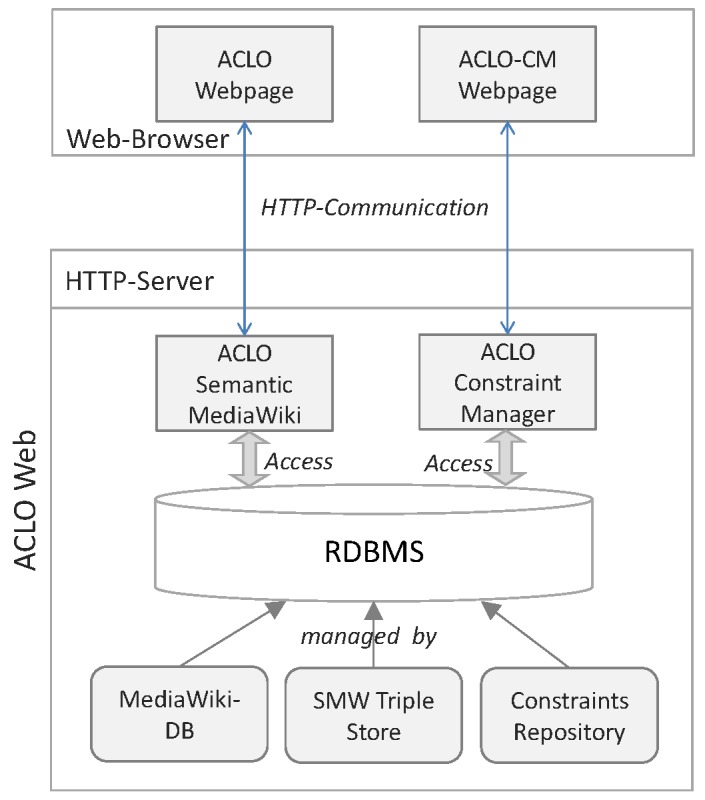
Architecture of ACLO-Web.

Therefore, ACLO-Web complements the SMW platform by adding a component enabling the definition and algorithmic check of enhanced consistency criteria (the ACLO consistency module or ACLO-CM). ACLO-CM is designed as a separate Web application operating on the SMW Triple Store and an additional constraint repository (Figure 3).

Consistency criteria are represented by expressions of the Object Constraint Language (OCL). Criterion C1, introduced above, is shown in Textbox 1.

ACLO-CM enables global consistency checking to be carried out at defined milestones. The component is not designed to prompt the users directly during data entry (as this would not be feasible for global constraints). Instead, it produces a detailed checklist, to be used for revision, of the problems found.

**Criterion C1.**

context curriculum inv: self.belong_to implies count(select(f|f.belongs_to.defined_in= self.belongs_to.defined_in and f.follows> size()=0))=1

### Formative Usability Study

A formative usability study assists the implementation of an SMW-based online catalog. Formative usability studies can be successfully carried out with only a few participants who can nonetheless indicate the most relevant usability problems. Nielsen recommended 5-8 testers [28].

We chose a scenario-based approach. The participants used the online catalog, sequentially solving tasks given in the context of a typical scenario that they were asked to imagine. We defined 7 imaginary scenarios involving both students and medical teachers:

- (S1) A student preparing for an examination and retrieving the learning objectives of courses recently visited
- (S2) A student interested in a topic because a relative suffers from a particular disease
- (S3) A student trying to gain an overview of the following term
- (S4) A student planning to specialize in a given medical field in the future, who is interested in where his or her chosen field is present in the curriculum
- (S5) A medical teacher preparing for a multidisciplinary course
- (S6) A medical teacher planning an examination
- (S7) A medical teacher trying to find out about the prior knowledge of the students enrolled in his or her course.

Data was acquired using an online questionnaire containing the textual description (vignette) of the scenarios and instructing the participants on the specific tasks for each scenario. After each scenario, the participants were asked to rate the following statements using a Likert scale: (1) “I could intuitively carry out all necessary actions”, (2) “I found all relevant information”, and (3) “There was too much redundant information”. Items 2 and 3 were accompanied by a free-text field where redundant and missing information could be further specified.

General feedback was acquired by 8 Likert-scaled items concerning (1) the relevance of the LOs retrieved, (2) the completeness of the results, (3) the performance of the system, (4) the feasibility of a meaningful interpretation of the results, (5) the comprehensibility of the labels used by the system, (6) the structure of the system’s output, (7) the design of the graphical user interface, and (8) the system’s usability in general. Finally, the participants could enter free-text feedback on positive aspects, negative aspects, and recommendations for improvements.

### Methods of Analysis

Due to the small number of participants, the scaled items of the usability test were analyzed by descriptive statistics only. The median, first quartile, minimal, and maximal values were derived from the data and visualized by box plot diagrams. The free-text feedback was analyzed by bottom-up qualitative text coding. Suitable keywords (codes) were produced and assigned to the text of the users’ statements while reading the text for the first time, the set of keywords was ordered and normalized, and then the keywords were used to index the statements consistently while reading the text for the second time. The statements were then rearranged by producing a synopsis of statements assigned to the same keywords and interpreted.

### Software

The online questionnaire for the usability study was based on LimeSurvey (v 1.85) [32]. Descriptive statistics and box plots were produced by R (v 2.14.0) [33]. Qualitative text analysis used Microsoft OneNote 2010. The online catalog was implemented on a virtualized Debian Linux-based XAMP server (v 1.8.0) [34] using MediaWiki (v 1.18.5 with SemanticBundle extension r20120327) [35].

## Results

### State of ACLO-Web

**Figure 4:**
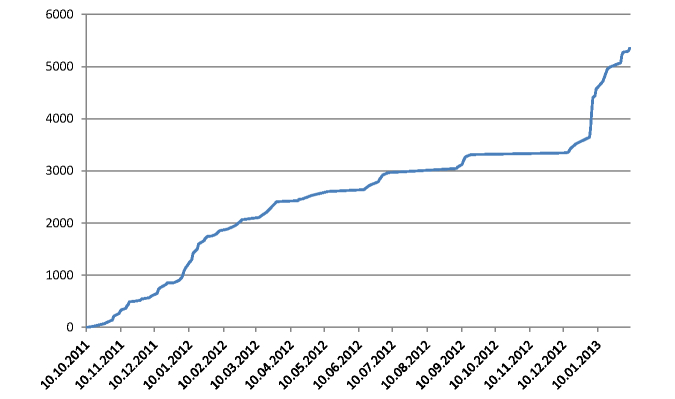
State of ACLO-Web: date vs total number of Learning Objectives acquired.

The online catalog of learning objectives is based mainly on 9 wiki categories (“Code/ID”, “Curriculum”, “Teaching Module”, “Learning Objective”, “Medical Learning Objective”, “Medical Specialty”, “Medical Topic”, “Segment”, and “Term”), 42 wiki templates, and 49 semantic properties of the SMW. The main activity concerning the data entry of LOs started in September 2011. By February 2013, 5350 LOs of the Aachen Medical Model Curriculum had been collected. The LOs were assigned to 69 modules, 61 medical specialties, and 243 medical topics that are defined in the system. Figure 4 shows the growth of the number of acquired LOs during this time interval.

### Lessons Learned During Implementation

The use of the free, open source SMW platform enabled a fast and low-budget implementation of ACLO-Web. A first version was available—and immediately used by the LO team—only a few weeks after starting the project. Therefore, the system (categories, attributes, and online forms) evolved during the implementation process of the catalog, a process that included several small modifications and one drastic change concerning the conceptual model. The latter resulted from improving the representation of different versions of the curriculum, due to changes in the local examination regulations (ER). We had to learn that, in different ER versions, existing LOs are included in different modules and whole modules may belong to different terms. Thus, some of the relations given in the core data model (Figure 2) needed to become dependent on the ER versions. Technically, this was achieved by introducing so-called semantic internal objects. The flexible way of imposing semantic constraints by semantic forms and templates (Figure 1) greatly supported schema versioning and content migration.

Additionally, for organizational reasons, a migration of ACLO-Web to different server hardware was necessary twice. In spite of an ongoing acquisition process, the system evolution went remarkably smoothly.

### Views and Queries Based on the Core Data Model

The online LO catalog provides different ways of viewing and retrieving LOs. In general, there are 2 methods of access: (1) browsing predefined overviews, and (2) searching by criteria individually defined by the user. The model introduced above grants 3 independent views: users can select and view LOs according to their position in the curricular context, their thematic focus, and the faculty and unit responsible for them, respectively.

**Figure 5:**
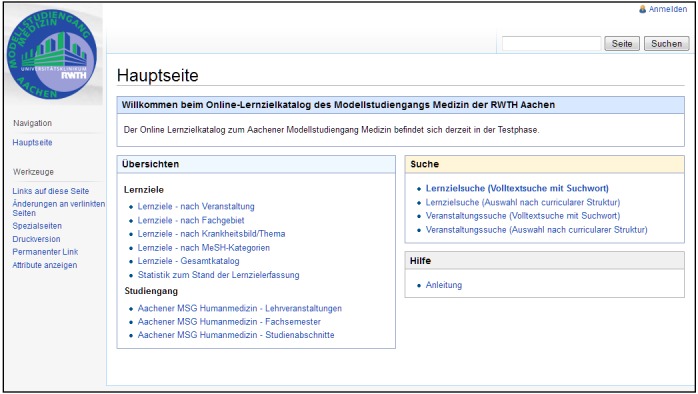
Main page of ACLO-Web.

Figure 5 shows the main page of the catalog. In the left-hand area, the page offers access via overviews, and in the right-hand area, the user can access search forms. All overviews are implemented as predefined searches (ie, overviews are not created manually, but instead are generated by the system using inline queries). Thus, all overviews are based on the actual state of the LO repository, and immediately show changes concerning added, modified, or deleted LO information.

In order to use a predefined overview, students and faculty members first choose a curricular module, a teaching unit, a medical topic, or a MeSH term. The system then shows an overview of the LOs associated with this entity in a tabular layout. Detailed information on a particular LO is accessible by following the Web link to the wiki page describing the LO (provided by the overview). All tables can be dynamically rearranged by clicking on the heading of one or more columns, indicating the sorting criterion. The default order presented first depends on the type of overview (Table 1).

The overviews based on medical topic and medical specialty provide a longitudinal projection, that is, the users can see at which point in the course of study the topic is addressed or the specialty is involved in the curriculum, respectively.

The forms for searching by criteria enable (1) the retrieval of LOs by selecting the respective building blocks/segments of the curriculum, ie, module, term, part of the study, or examination regulation, and (2) a full-text search (supported by the auto-completion of terms used in the description of LOs).

**Table 1:** Default ordering corresponding to different types of LO overviews.

Overview of concerns	Sorting categories
Curricular module	Order in which LOs are addressed in the module
Medical specialty responsible for LO	Module associated with the LO (following the course of the curriculum)
Medical topic	Module associated with the LO (following the course of the curriculum)
MeSH based LO category	Short title of the LO (in lexicographic order)

### Collaborative Specification of Learning Objectives

As a key feature of the Social Semantic Web-based approach, the online catalog allows a distributed, collaborative, and structured data entry, requiring the client to have nothing more than a Web browser (and of course an Internet connection). The wiki-based user interface uses 13 online forms for data entry and 4 online forms for flexible LO search.

**Figure 6:**
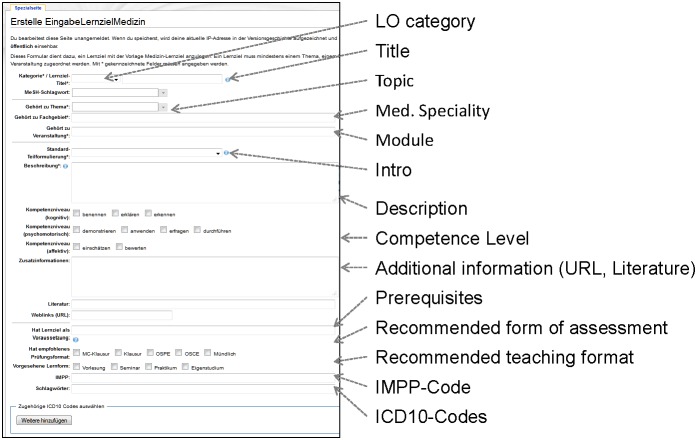
Form for defining a medical learning objective.

Figure 6 shows a screenshot of the form for describing LOs. Due to the high number of input widgets and the German labels of the system, the screenshot comes with additional English labels. The attributes used for specifying an LO have been introduced in the methods section above. In order to support offline data collection, we equipped the representatives responsible for the LO specification with predefined Excel forms that they could alternatively use for specifying LOs. The LO specifications were afterwards transferred to the system by the LO catalog team, and then checked online by the representatives.

Following the elicitation process described above, primary data entry was centralized and carried out by the ACLO team in order to foster high and uniform data quality, and consistent semantic indexing. The faculty was then encouraged to revise the catalog. Following the wiki approach, ACLO-Web avoids the fine, granular access restrictions that are present in many content management systems. Thus, versioning support and page comments offered by the platform play a pivotal role in supporting an open, but nonetheless socially controlled, revision process.

### Global Consistency Checking: ACLO-CM

**Figure 7:**
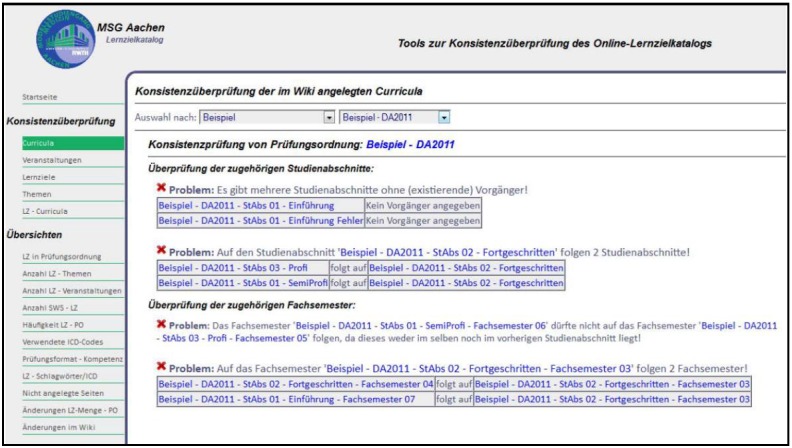
Report on global consistency problems generated by the ACLO-CM component.

Figure 7 shows a screenshot of the component for checking global consistency criteria (ACLO-CM). At present, ACLO-CM defines and checks 12 OCL constraints. Additionally, ACLO provides a convenient overview of modified SMW entries, which is structured by the categories of the model (see Figure 1). The report on existing consistency problems generated by ACLO-CM is structured by the categories of the affected SMW entries and the criteria checked. Figure 7 shows a typical ACLO-CM report.

### Formative Usability Study

**Figure 8:**
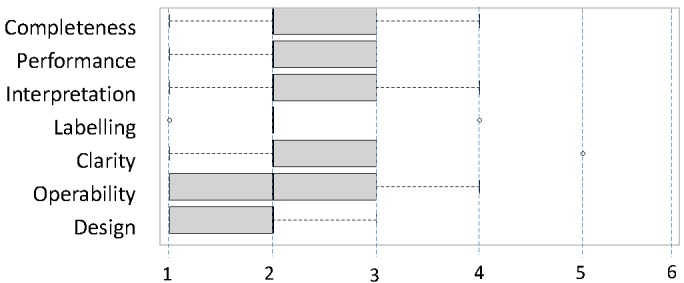
Overall rating of usabililty aspects (Rating scale: 1 = “Very good” to 6 = “Unsatisfactory”).

The formative usability study was carried out in August 2012. Five medical students (in their second and third years of study) volunteered to participate in the study. Figure 8 shows the results of the overall rating of usability items after completing all 7 scenarios. The qualitative analysis of the full-text answers yielded 30 separate statements assigned to 17 codes by the analysis.

Table 2 gives an overview of the code categories, codes, and numbers of statements assigned. Particular usability problems were raised in 10 statements, which addressed problems with the navigation and orientation (3 statements), comprehensibility, especially the interpretation of the short title of the LO (3 statements) (4 statements in total), and the presentation of the LO description (3 statements). These last statements said that the LO presentation should avoid the repeated presentation of the standard introductory text and should integrate the competence levels (verbs) into the textual description of the behavior.

With respect to the actual state of the LO repository, missing LOs were reported; some participants named missing aspects in detail (5 statements). The participants found very similar LOs repeatedly addressed by different medical specialties (4 statements), were not able to find a suitable starting point for their search in the case of scenario S5 (2 statements), and—concerning the same issue—suggested the introduction of an attribute indicating multidisciplinary teaching formats (2 statements).

The statements contained 5 detailed recommendations, including the one proposing the attribute for multidisciplinary teaching formats. The extended search forms were recommended (2 statements); one participant suggested a graphical timeline for visualizing the longitudinal distribution of LOs.

The participants explicitly encouraged the alternative ways of accessing the LOs by overviews (1 statement), the structured, table-based presentation of the results (2 statements), and the longitudinal projection of a topic in the curriculum.

**Table 2:** Qualitative feedback: overview of the code categories derived from the statements.

Code category	Code	# of statements
**Navigation/Orientation**		
	Confusing SMW links	2
	Navigation/orientation problem	1
**Comprehensibility**		
	Interpretation of LO short title	3
	Interpretation of MeSH category	1
**LO presentation**		
	Competence levels as text	1
	Redundant introductory texts	2
**State of the catalog**		
	Missing LO	5
	Similar LO addressed by different specialty	4
**Insufficient support for search**		
	Unclear starting point of the search	2
	No index for multidisciplinary modules	2
**Recommendations**		
	Extended search	2
	Timeline view	1
**Encouragement**		
	Alternative views/ways of access	1
	Longitudinal view	1
	Structured, table-based overview	2

## Discussion

### Principal Findings

The focus of this paper is to report on the application of Social Semantic Web technology to support the knowledge management process and to enable the implementation and collaborative maintenance of an online catalog of learning objectives of a complete medical curriculum. Thus, we address neither the issue of the effect of competence-based learning objectives on the learning outcome nor a summative evaluation of the quality of ACLO.

Nonetheless, we report the results of the formative usability study because they were an essential part of the implementation process. Although it is known that usability studies can successfully rely on a relatively small number of participants [36], in our case the number is at the low end even for a formative study. The selection of the participants may be further biased, because we asked the official medical student representatives to name students who would possibly volunteer to participate in the study. Thus, the participating students can be assumed to take more interest than average in the further development and improvement of the existing model curriculum. The study did not involve the faculty. Although a medical expert was part of the LO team, his feedback was continuously incorporated into the implementation process. Nonetheless, the usability study will have to be extended to faculty members in the future.

### Implications of the Usability Study

Taking into account the limitations mentioned above, the usability study nonetheless produced extremely valuable feedback that led to the improvement of the online catalog. The participants gave detailed hints on problems concerning orientation within the catalog, comprehensibility, and performance of the system. The hints on navigation problems and the interpretation of the short titles have already led to improvements in the present version of the system. Confidence in the results of the study is increased by observing a marked accordance of quantitative and qualitative feedback (triangulation); the overall rating of usability aspects yields good results (especially with respect to operability and design), while some participants (voluntarily) encouraged the approach (especially complimenting the flexible, structured overviews). The main criticism concerned not the application but the state of the catalog, which was indeed poor when the formative evaluation took place and has markedly improved since. The box plots yielded a low rating for the completeness of information. In the context of the same scenario, the qualitative feedback stated that information on multidisciplinary modules was missing, or proposed an additional wiki attribute for capturing the relevant information.

As a main implication, the study showed that the online catalog actually works as an instrument for quality improvement and communication concerning LOs; while looking for different LOs assigned to the same medical topic, the students detected very similar LOs that were specified independently by different specialties. These require further coordination and reconciliation. The authors did not know about these findings during the preparation of the scenarios.

### Enabling Nonproprietary Solutions

As noted in the introduction, custom-built software dominates the field of curriculum management systems. The SMW-based approach fills the gap between individual and flexible requirements and existing software platforms for supporting curriculum management. The fact that a first version of ACLO was ready for production use only a few weeks after the start of the project indicates that the SMW platform enabled the developers to easily configure a system that fulfills the specific requirements of a given CM project and supports the needs of different users. Furthermore, the data model and the information content of ACLO are accessible via standardized interfaces (eg, SPARQL queries) and can be exported to the W3C-standardized OWL format. Both aspects foster semantic interoperability and cross-platform migration. From a technical perspective, the availability of the MediaWiki Web application programing interface (Web-API)—well known from Wikipedia—seems even more important. The Web-API requires nothing but a Web connection in order to enable other systems to interact with ACLO.

The available formats and interfaces of the SMW-based approach not only improve standardized information access and system interoperability, but also allow importing existing classifications or ontologies to the system. This feature enabled, for example, the import of the ICD into ACLO. Furthermore, the system’s information content can be easily enriched by seamlessly including interwiki links to other wiki-based information sources including Wikipedia.

Overall, the SMW-based approach shows potential to provide a versatile, but partly standardized platform for curriculum management, especially supporting collaborative aspects—far from being “yet another IT tool”.

### Comparison With Prior Work

As stated in the introduction, ontologies serve as a means for semantic indexing [21-24]. Semantic indexing in ACLO uses not only medical standard classification, but also a folksonomy—following the collaborative approach. If compared with existing second-generation curriculum management systems such as eMed [18] and CMap [20], ACLO does not yet offer the complete functionality found there; neither the management of learning portfolios nor the support of all administrative planning tasks (eg, the management of timetables or resources) are presently supported. In contrast, none of these systems profits from the semantic standards and collaborative approach of the Social Semantic Web.

The Semantic Web has long been discussed as a means for supporting curriculum development by representing the learning design and content of a curriculum [37]. Following this line of tradition, Tang and Rahman developed a system design, which combines an ontology of the domain covered by a curriculum (here, computing domain) with a wiki-based information system [38], and Segeninac et al proposed to apply Semantic Web technology to curriculum development by modeling metadata for learning opportunities [39]. Recently, Coccoli et al outlined the potential of semantic wikis for collaborative curriculum development [40]. None of these system concepts or demonstrators made its way to productive use and none was intended to support curriculum management in the field of medical education. To the best of the authors’ knowledge, ACLO is pioneering as a medical curriculum management system rigorously based on a Social Semantic Web approach and platform.

### Agile Knowledge Management by SMW

The growth of the number of LOs during the past year shows that the system successfully supports the elicitation and systematic collection of LOs. As shown in Figure 4, the environment successfully supports an active process of the elicitation of learning objectives. The rapid increase of the number of LOs during the past months can be explained by the preparation and announcement of a faculty-wide evaluation process; this has obviously operated as an incentive. We received positive feedback concerning the applicability and usability of the system, not only from participants in the usability study but also from the LO team and faculty involved. This may be an effect of the familiarity of nearly all users with Wikipedia; the use of the MediaWiki platform seems to have led to a low threshold for using the LO catalog.

At present, ACLO uses the few user roles originally provided by the MediaWiki platform. Thus, all members of the faculty can edit the LOs contained in ACLO during the revision process. This fact is causing controversy—part of the faculty demands the introduction of fine-grained, role-based access control, while others advocate the existing approach, relying on versioning support, the transparency of the page history, and shared social media etiquette.

Kiessling et al report on a systematic consensus process that improved the definition of (outcome-based) learning objectives, which the authors consider an intrinsically collaborative process [41]. Their statement is in line with the finding that curriculum management is resource-intensive and requires systematic change-management [9]. Additionally, Wong and Roberts argued that the procedural nature of curriculum mapping requires ongoing IT-enabled feedback [2]. Wikis proved to enable complex consensus processes and collaborative planning in the medical domain [42]. We consider that the SSW-based (Web 3.0) approach is an ideal platform for enabling and efficiently supporting curriculum mapping. The SMW platform, its versioning support, and standardized import/export formats effectively enabled successful evolution of the system. The weak schema constraints imposed by the approach (Figure 2) allowed a very flexible evolution of the model, while at the same time enabling structured information management and access.

Thus, the SMW-based approach proved to enable an agile implementation of computer-supported knowledge management. The approach, based on standard Social Semantic Web formats and technology, represents a feasible and effectively applicable compromise between answering to the individual requirements of curriculum management at a particular medical school and using proprietary systems. Given the overall feasibility of a Web 3.0-based curriculum management system, these special aspects of a flexible and agile knowledge management can indeed foster collaborative curriculum mapping as a feedback-driven process.

### Future Directions

As mentioned before, further formative usability testing and the scheduled revision phase will involve all members of the faculty. ACLO will be open for all students at our medical school in approximately April 2013. We are now preparing a summative evaluation of the system, including a log file analysis of the users’ behavior. At present, 2 questionnaires (addressing students and faculty, respectively) will undergo pilot testing and a revision process. The questionnaire for faculty contains scaled items concerning qualitative aspects of the learning objectives’ specifications (SMART-criteria: Specific, Measurable, Accepted, Realistic, Timely), the completeness, and the perceived effectiveness of the catalog. The students’ questionnaire further addresses the students’ appraisal of the benefit from ACLO. A second curriculum (dentistry) is going to be included in 2014.

Maloney et al showed that medical students appreciate the benefit of online repositories of learning resources [43]. They also showed that milestones of the curriculum (eg, examinations) often trigger access to the repositories. Consequently, ACLO will be integrated with an already existing system used for the consistent indexing of eLearning media, by a semantic network based on the Medical Subject Headings and ICD codes, which were linked by associations taken from the SNOMED Clinical Terms terminology. The integration will link the topics and external vocabularies associated with ACLO learning objectives to concepts of the semantic network and will therefore extend the systems capability of finding similar LOs.

Last but not least, the ongoing project of a German national catalog of competence-based medical learning objectives will heavily influence the future development of ACLO-Web and result in challenging tasks concerning system integration and LO identification. This will eventually be based on ontology mapping approaches.

## Abbreviations

ACLO: Aachen Catalogue of Learning Objectives
ACLO-CM: ACLO consistency module
ACLO-Web: Web-based version of the Aachen Catalogue of Learning Objectives
AMMC: Aachen Medical Model Curriculum
ER: examination regulations
HSDB: Health Science Database
ICD: International Classification of Diseases
IMPP: Institute for Medical and Pharmaceutical Examination Items
LO: learning objective
MeSH: Medical Subject Headings
OCL: Object Constraint Language
OPS: German modification of the International Classification of Procedure in Medicine (Operationen- und Prozedurenschlüssel)
OWL: Web Ontology Language
SCLO: Swiss Catalogue of Learning Objectives
SMW: Semantic MediWiki
SSW: Social Semantic Web
